# Going Above and Beyond: Bridging the Gap Between Equitable Outcomes and Procedural Fairness in Health Policy Administration

**DOI:** 10.3390/healthcare12232427

**Published:** 2024-12-03

**Authors:** Danielle N. Gadson, Seri Park

**Affiliations:** 1 Department of Public Administration, Villanova University, Villanova, PA 19085, USA; 2 Department of Civil & Environmental Engineering, University of Nevada, Reno, NV 89557, USA; serip@unr.edu

**Keywords:** health administration, health equity, public health policy, opioid epidemic

## Abstract

Background/Objectives: Public health administrators are entrusted to oversee the fair and efficient implementation of public health policy. Professional standards rooted in social justice add an additional ethical standard beyond what is required by procedural equality, reinforcing a service culture of creativity and doing more with less when resources are restrictive. This study explores this phenomenon within the context of government-subsidized opioid use disorder programming in Pennsylvania. Methods: Twenty-three agencies responsible for opioid treatment referrals in Pennsylvania were surveyed about the effects of meeting social equity needs on their operational and procedural outcomes. Univariate frequencies, cross-tabulations, and percentage comparisons were employed in a descriptive–analytic induction approach to analyze the online survey responses. Results: The survey results showed that 91.4% of administrators identified transportation equity as an important programmatic concern, with 91.3% developing transportation support for their clients although not required or funded by the grant program. Conclusions: Equity-focused interventions necessitated operational creativity and sacrifice to maintain compliance while meeting the unique needs of populations, especially when taking geographical differences into account.

## 1. Introduction

Public health policy administration in the United States is rooted in a social and political ideal that citizens have a constitutional right to fair and equal access to public resources, and everyone should have the same opportunities for success in our society regardless of the social groups to which they belong [1,2,3]. The public entrusts public administrators to implement and manage public policy initiatives fairly and justly to ensure the effective distribution of resources, as social equity has long been identified as a programmatic goal in public policy [4,5,6]. Public administrators are uniquely positioned to fairly and equitably redistribute public resources to mitigate the effects of historical inequities whenever feasible; this is especially true regarding healthcare and when addressing health disparities. However, an administrator’s well-intentioned attempt to achieve health equity can be at odds with the traditional bureaucratic structure from which programmatic requirements are initiated. Because public health initiatives funded with public dollars tend to have restrictive implementation rules, fulfilling a professional responsibility to socially equitable outcomes often requires cultivating an organizational culture that prioritizes creativity and sacrifice over convenience and agility in implementing programmatic solutions. In this sense, contemporary public administrators are challenged to meet the unique needs of their communities and prioritize socially just outcomes while balancing efficiency and effectiveness in program implementation. The responsible administrator guides their agency toward social equity and justice in public policy administration, even when professional accountability may only require procedural fairness in the distribution of healthcare resources [7,8].

This study explores how public health administrators navigate this challenge within the context of opioid addiction treatment in state-sponsored programs. The problem of opioid addiction and associated death rates has been a persistent concern for public health agencies for several decades. In 2021, an average of 45 people suffering from opioid use disorder (OUD) died per day in the United States because of their addiction. Since 1999, the total number of opioid overdose deaths has topped three-quarters of a million people across all social demographics [9]. The categorization of opioid use addiction as a national epidemic by the U.S. Surgeon General and the Department of Health and Human Services unlocked federally appropriated federal funds totaling nearly USD 9 billion since the beginning of 2017 to comprehensively address OUD prevention, treatment, and recovery across every community. The policy response to the crisis has been a mixture of legislative actions, annual appropriations for federal agencies, and competitive state-level grants managed by the Substance Abuse and Mental Health Services Administration (SAMHSA) [10]. However, despite these earnest efforts, opioid addiction and death rates have continued to rise until recently, especially among individuals living in counties with high rates of income inequality, non-Hispanic Black males, American Indian or Alaska Native persons, and individuals living with untreated mental disorders [11,12].

### The Public Health Approach to Treating Opioid Use Disorder

Although initiated at a federal level, public OUD funds are primarily administered at the state or local levels by state public health agencies using a combination of Medicaid reimbursements and competitive grants. Central to this strategy is the enrollment of eligible patients into programs combining medications for OUD (MOUD) with mental and behavioral healthcare and social support on an outpatient basis. This evidence-based approach to care has generally been successful in the reduction in opioid dependency in clinical studies; however, the efficacy of the intervention is variable and follows common stratification patterns based on social identity and factors such as race, socioeconomic status, and geographic location [13,14,15,16]. Like the outcomes of other disease conditions, those with fewer social barriers to care tend to fare better, while those with disparate access to care fare much worse with OUD treatment outcomes in the long run. Moreover, the opioid crisis has been particularly crippling in rural areas where residents perceive drug addiction as the most pressing problem in their communities, closely followed by insufficient access to public transit systems [17]. Opioid death rates are particularly troubling in Pennsylvania, where the rate was twice that of the national average in 2017 [18].

Despite the national attention, the proportion of individuals suffering from OUD and actively in treatment is relatively low [19]. With more than 40% of individuals suffering from OUD enrolled in Medicaid, public insurance coverage is a critical component of the federal government’s efforts to provide early intervention and treatment of OUD in the United States. All state Medicaid programs cover at least two MOUD medications; however, as of 2019, just 31 states covered outpatient detoxification [20]. This poses a barrier to treatment, especially for those without reliable access to transportation. The impact of Medicaid expansion under the Affordable Care Act (ACA) on OUD treatment access is significant and promising, enabling the state to provide more social services, including detoxification, psychotherapy, peer support, employment programs, supportive housing, job coaching, and peer recovery coaching for adults with income up to 138% of the federal poverty level. As of 2024, 41 states have adopted ACA expansion, including Pennsylvania. ACA coverage will likely become increasingly restricted under President Trump’s second term.

Paradoxically, MOUD regimens are inflexible by design. Patients must receive medication on a strictly supervised and rigid schedule, undergo periodic random drug testing, and maintain regular contact with their medical support team. Treatment plans are patient-specific and consider factors such as addiction severity, patient preference, insurance status, and proximity to treatment facilities. In all instances, an individual’s treatment plan highly depends on their access to reliable and timely transportation. Patients can be in care for months, years, or even a lifetime. Evidence shows that primary care providers (PCPs) are best positioned to manage these treatment programs; however, continuity can be an issue due to transportation challenges, especially in rural areas [21].

Because the current standards of OUD treatment depend on these frequent interactions with the treatment team, equal and equitable transportation is a critical factor in the equitable implementation of OUD treatment programs. In 2022, over 15 million Americans reported having unreliable transportation [22]. Across all health conditions, poor patient transportation options often result in delayed or missed health appointments, increased health costs, and overall deteriorated health outcomes [23]. The challenge of transportation inequity complicates the rollout and implementation of public health initiatives on a local level. This study explores how administrators of government-funded OUD treatment initiatives merge a professional responsibility to promote social equity with the limited resources provided by public health agencies to carry out their work. The findings support the hypothesis that federal subsidies are often insufficient to appropriately address the equity needs of communities, requiring local administrators to impart significant creativity and sacrifice to meet their professional responsibility for social equity. Using the transportation challenges of public OUD treatment initiatives in Pennsylvania as an example, we offer concrete recommendations to align equality and equity in public programs. This study aims to bring attention to the unique burden placed on public health administrators to achieve social equity outcomes when program rules and funding stop at the requirement of equality. This would negatively affect the efficacy of public health policy initiatives if it were not for the flexibility, creativity, and commitment of public health administrators and their teams to meet the unique needs of the communities they serve.

## 2. Materials and Methods

### 2.1. Study Design and Participants

Like many other states, Pennsylvania has deployed a specialist-based referral model to help Medicaid-eligible populations suffering from OUD access care, where those individuals identified as needing support are handed off to authorized public agencies specializing in referring patients to the appropriate care resources based on their demographics and personal circumstances. The Centers of Excellence (COE) initiative was introduced in 2016 to activate federal funds to triage and treat Medicaid-eligible individuals in the state suffering from OUD. Combining long-term medication regimens with a network of non-clinical agency navigators to coordinate care, the agency administrators manage the program using standardized guidelines established at the state and federal levels. These non-clinical agency navigators play a crucial role in the COE program, as they can help address transportation challenges by coordinating and arranging transportation for clients. A signature component of the COE program is the face-to-face preliminary assessment and then warm hand-off of clients to treatment providers within a preauthorized network. As of 2020, 45 COEs served over 32,500 Medicaid-eligible Pennsylvanians [24].

The primary research method for this student was an online survey distributed to public health administrators working at COE agencies in Pennsylvania. The survey tool presented agency representatives with a series of questions to understand how transportation barriers might influence how agencies refer individuals suffering from OUD into care. More specifically, the survey aimed to illuminate how social factors such as geographic location and individuals’ access to transportation can influence how agencies equitably administer COE resources that the federal government intended to deploy uniformly. To develop the email list, the Pennsylvania Department of Health website was used to identify and collect the contact information of administrators at COE-designated agencies. Following confirmation of IRB exempt status, we sent introductory emails to all COE agency contacts to solicit their interest in participating in a brief electronic survey on transportation equity in opioid use disorder treatment in Pennsylvania. The introductory email explained that the study aimed to collect agency reflections on the influence of disparate access to public and private transportation options when referring Medicaid populations to OUD treatment and care. We also reassured agency representatives through email that their responses would be kept confidential and that their agencies would remain anonymous. After several solicitation rounds, 24 of the 45 COE agencies agreed to take the survey, representing a 53.3% response rate.

### 2.2. Data Collection and Tools

An electronic survey was designed by the research team utilizing Qualtrics XM version June 2022. The core survey comprised three general sections: demographic information about the agency and its clients, questions about transportation challenges in referring OUD clients for treatment, and the reporting of agency-specific solutions for overcoming any challenges. There were 18 core demographic questions of various types, including rankings, Likert scales, and self-reported numerical estimates. Surveys with multiple land use types or service sites repeated some core questions to allow for site-specific variances. The data from the responding sites were anonymized and coded for the land use type (urban, rural, and urban cluster) and significant themes related to program implementation and transportation challenges. To develop and validate the survey questions, informational in-depth interviews were held with five stakeholder agencies in a preliminary stage of the study to identify potential topics and trends that may be relevant to the survey process.

Before deployment, the research team used public information to categorize the 24 participating agencies into survey groups based on the number and location of their service sites using the PA Department of Health website, the 2010 Census Urban and Rural Classification definitions, and location zip codes. The categorizations were then used to deploy one of three nearly identical electronic surveys via Qualtrics XM to an agency administrator: (1) single service site, (2) multiple service sites with the same urbanicity category, or (3) multiple service sites across different urbanicity categories. The three surveys varied only to allow variations in data reporting based on location type with minimal redundancy of agency-level demographic responses. Table 1 outlines the structure of each survey variation.

Once the agencies were categorized by survey version, the single service site version of the survey was distributed to 16 of the 24 agencies. The multiple service sites across different urbanicity category version of the survey were distributed to 7 of the 24 agencies. The numerous service sites across different land use categories version were deployed to just one agency. All but one of the agencies that agreed to take the survey completed it in full. The incomplete survey was one of the seven surveys deployed under the multiple service sites with the same urbanicity category for all site versions.

### 2.3. Statistical Analysis

Univariate frequencies, cross-tabulations, and percentage comparisons utilizing Qualtrics XM version December 2023 and Microsoft Excel’s version 2410 analytics function were employed in a descriptive–analytic induction approach to assessing potential trends applicable to the sample. Due to the limited sample, only descriptive trends for the sample are reported and discussed in this study. Chi-square tests were performed but were insignificant with a *p*-value set at <0.05, and were thus excluded from the tables.

## 3. Results

In total, 23 out of the 45 COE-designated agencies in Pennsylvania fully completed the online survey, representing 16 counties of various geographical landscapes. Overall, 9 of the 23 agencies operated sites in urban areas (39.1%), 7 operated sites in rural areas (30.4%), and 7 operated sites in suburban areas (30.4%). Half of the agencies had their COE designation for two years or less, and nine had their designation for more than four years. While all responding agencies offered opioid treatment referrals, case management, and counseling services to Medicaid-eligible individuals suffering from OUD, additional agency services included behavioral healthcare (46%), primary healthcare (29%), prenatal care (17%), and access to certified or peer recovery specialists (17%). Nine agencies (39.1%) specifically listed the MOUD regimen as a primary service. Most agencies reported servicing 100–500 clients annually (67.5%), with the volume ranging from less than 100 (12.5%) to more than 500 (21%).

Table 2 summarizes responses to the first of several survey questions about transportation equity and its effects on program implementation. The first set of responses details the importance of considering transportation needs when referring individuals to care. In total, 82.6% of responding agencies indicated that transportation considerations are “very important” or “critical” when referring or enrolling individuals with opioid use disorder into care. This percentage slightly increased to 85.1% for agencies operating in a suburban area and to 100% for agencies operating in rural areas. The consideration of transportation needs was more varied for those operating in urban environments, with 22.2% of agencies indicating that it was “slightly important” or “not at all” influential, with another 11.1% of agencies characterizing the issue as “important” and the remaining 66.67% reporting it as “very important” or “critical” when making referrals or enrolling individuals with OUD into care. Relatedly, survey respondents were asked to estimate the percentage of OUD treatment-related appointments missed per week due to transportation barriers. Responses ranged from 5% to 50%, with an average of 23% of scheduled OUD clients missing appointments.

**Figure 1:**
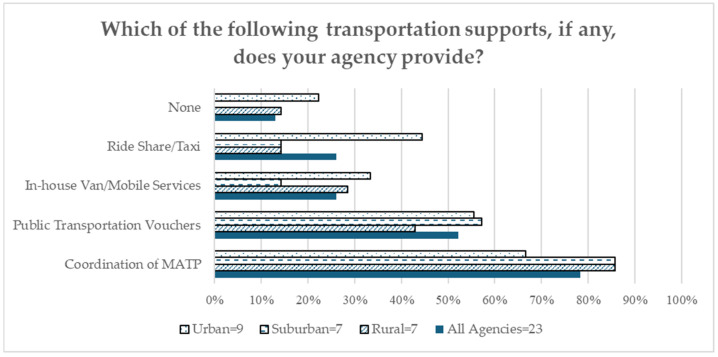
COE Transportation Equity Survey: transportation support (all agencies = 23; rural = 7; suburban = 7; urban = 9).

Figure 1 lists the transportation support agencies that reported helping clients to mitigate the threat of missed appointments due to transportation challenges. In total, 21 of the 23 agencies reported offering some transportation support to individuals, with the most commonly identified being MATP coordination (78.3%), the offering of public transportation vouchers (52.2%), and the remission of private car-share services such as Uber or Lyft (26.1%). In total, 6 of the 23 agencies reported having in-house van services, but several noted the service as having “very limited capacity”. The coordination of MATP services was more likely in rural and suburban areas, and public transportation vouchers and ride-share/taxi coverage were more likely in urban settings. Suburban locations reported the lowest availability of in-house van or mobile services.

Despite the transportation equity challenges and their associated costs, most agencies expressed satisfaction with the level of funding provided by the state and federal government. While local administrators had some financial flexibility in planning and using awarded funds in the program’s early days, in 2019, the COE funding switched from grant-based to a Medicaid per-member-per-month rate. With the change, the same flat rate of USD 227.22 per member per month was paid to COE agencies for face-to-face care management, regardless of the location or unique needs of the client population. Notably, there was no special consideration for transportation costs in the rate despite known risks of transportation inequities based on geography. The state’s Medical Assistance Transportation Program (MATP) does, however, provide county-specific transportation to medical appointments for medical assistance recipients with limited transportation access. The MATP emerged as a major resource for moving OUD patients into care in this study. As shown in Table 3, all agencies reported that the blanket COE per-member-per-month rate funds all components of their OUD treatment activities slightly well or better, except for one agency operating in an urban environment.

Table 4 illustrates that according to the self-reporting of agencies, not all patients are perceived to have difficulty accessing OUD care. While most agencies indicated that it is ‘difficult’ for their OUD treatment clients to attend their treatment-related appointments, this was mostly driven by the agencies serving in rural and suburban locations. In total, 4 out of 23 agencies (17.3%) reported it was ‘easy’ or ‘extremely easy’ for clients to access treatment appointments. The geographical location of the agencies made a difference here, with suburban agencies being most likely to report that their clients find it difficult to attend their appointments (71.4%), compared to 57.1% of rural agencies and 44.4% of urban agencies.

**Figure 2:**
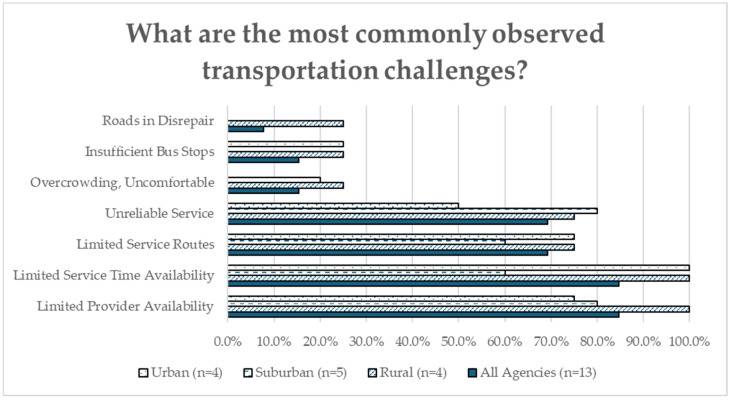
COE Transportation Equity Survey: commonly observed transportation challenges (all agencies = 13; rural = 4; suburban = 5; urban = 4).

Figure 2 examines the 13 out of 23 agencies that reported it was difficult for their clients to attend treatment-related appointments. Nearly all agencies in this subset pointed to a limited number of transportation service providers (84.6%) or limited service time availability (84.6%) as top concerns impacting transportation equity. Other common barriers were limited service routes (69.2%) and unreliable services (69.2%).

**Figure 3:**
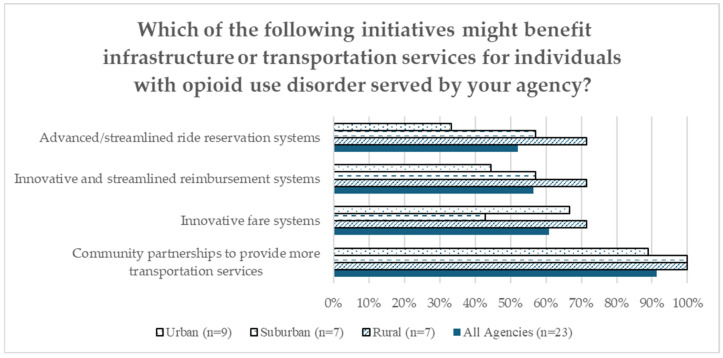
COE Transportation Equity Survey: proposed improvements (all agencies = 23; rural = 7; suburban = 7; urban = 9).

As a final question, agency respondents were asked what type of initiatives might benefit transportation infrastructure or services for the individuals served by state-sponsored OUD treatment programs. Figure 3 displays the top responses. Respondents overwhelmingly identified partnerships with other community organizations as an essential intervention, with 22 out of 23 respondents indicating this as beneficial. Other recommendations were innovations to fare systems (61%), innovative and streamlined reimbursement processes, and advanced and streamlined ride reservation systems (52%).

## 4. Discussion

The findings of this study reinforce well-documented challenges of transportation equity in the treatment of opioid addiction through the lens of the organizational partners that coordinate resource access for vulnerable populations. While the development of public health initiatives is traditionally steeped in the ideal of equal access to healthcare and health resources, this study’s findings emphasize that vulnerable populations served by public health initiatives are not a homogenous demographic. In the case of opioid addiction and treatment, the personal circumstances and community characteristics of the individual undergoing care significantly impact that individual’s ability to effectively seek care, find suitable treatment programs, and stay compliant with those programs. Even for the most willing participant, successful completion of an OUD treatment regimen is highly dependent on access to quality services and reliable transportation. MOUD regimens are most effective when coupled with behavioral and mental health interventions and social support. This requires the administration of a strictly controlled medication on a supervised and rigid schedule, coupled with frequent face-to-face check-ins with a multi-specialty team of clinicians, social workers, and peer support, translating into weekly, if not daily, face-to-face clinic interactions, even for outpatient care. The required schedule can be particularly taxing for individuals living in geographic areas with disjointed or insufficient transportation structures.

The findings also point to the variations in the impact based on geographic location as defined by land use categories. Agencies operating in urban areas reported easier client access to transportation options than agencies operating in suburban and rural areas. This finding was generally expected as in the pilot stage of this study consisting of informational interviews, agenda leaders in urban environments explained that treatment appointments can be spread all over the city, making the cost of transportation very high as clients need to pay for multiple rides in a short period. Agencies operating in rural environments spoke of fewer transportation options and longer travel distances between home and treatment locations, making transportation coordination and schedules difficult, especially if the individual cannot access a car. Even when the in-house van service was an option, its use was limited. Offering in-house van transportation or coordinating paratransit services requires significant human resources beyond any financial resources associated with vehicular costs or reimbursements, as meticulous scheduling is needed to ensure that clients do not have unnecessarily long wait times for drop-offs and pick-ups. Also, service hours can be challenging and expensive as a van schedule adhering to traditional working hours can limit the number of individuals receiving service, especially when operating in an area with a broad geography. Restrictive provider office hours or a limited number of vehicles that can transport clients can exacerbate these barriers. Suburban challenges appear to be a hybrid of rural and urban operation locations. Public transportation is often available but disjointed and hard to access, requiring abnormally long bus rides or wait times for van services. When agencies offer in-house van services in addition to fares, they must maintain a significant budget for both public transportation outlays and the maintenance and upkeep of private vans. In the next stage of our study, we plan to conduct focus groups and interviews to better understand these qualitative differences.

For responsible public health administrators, programmatic success goes beyond equality for public health administrators, who are leading agencies accountable for the fair and equitable distribution of public resources. This study’s data underscore public health administrators’ dedication and effectiveness in mitigating transportation challenges. For example, several agencies named partnerships with local non-profits that provide free rides to individuals who have a substance use disorder as an example of these partnerships, demonstrating that this work is already occurring to some degree and indicating potential success for others to achieve the same. These data provide a sense of optimism about the likely success of such partnerships in improving transportation challenges for clients.

The equitable distribution of public services reflects a more profound commitment to correcting historical and social ills whenever feasible. This need is most pronounced for agencies serving individuals in rural or suburban areas due to the lack of transportation infrastructure and services. COE agencies operating in rural and suburban areas must absorb the impact of this additional challenge, as their budget allocations are the same as agencies located in urban areas with many transportation options. This absorption requires operational creativity and an organizational culture of doing more with less, a culture familiar to many public agencies. To meet this challenge, public policy guidelines must be flexible enough to foster equitable solutions to disparities that disproportionately hinder the progress of some social groups, such as racial minorities, individuals experiencing poverty, and those living in rural communities. However, this is not often the case for public health initiatives serving a broad swath of the community.

The results from this study underscore the need for flexibility in health policy initiatives in order to tailor resources to the variable needs of vulnerable populations. Expanding traditional explorations of these barriers to include an understanding of how agencies adapt to this challenge provides critical insight into how the public agency administrator commits to promoting social equity through their work. Nearly every agency reported that transportation was necessary when referring or enrolling Medicaid enrollees with OUD for treatment. Additionally, most agencies reported providing transportation support to mitigate the effect of this barrier effect for their clients, even though the function of transportation is not specifically reimbursed or required as a part of the grant. This operational sacrifice demonstrates administrators’ dedication to managing transportation challenges despite the additional operational costs and is a testament to their commitment to their clients’ well-being.

This study has several limitations, primarily the lack of generalizability due to the low sample size and the restriction to agencies operating in the state of Pennsylvania. For future research, a nationwide survey could significantly increase the sample size and improve generalizability, providing insight into whether the identified trends persist across geographical locations. Another limitation is that there was little investigation of the structural elements influencing transportation equity, such as public transit initiatives. Providing more of a case study investigation of the built and geography elements would add invaluable context for the study findings. Finally, it is important to note the change in the OUD treatment landscape since the survey data were collected. MOUD restrictions have been softened from a federal perspective, making medications more accessible and removing some stigma from MOUD programs and initiatives. Repeating this study in this new and consistently improving environment may yield variable results from this study. The findings could also be enhanced by incorporating a qualitative component to the study. In future iterations of this work, we plan to interview and hold focus groups with clients and agency representatives to obtain a more detailed description of the administrative challenges.

## 5. Conclusions

This study aims to raise awareness of the distinction between equality and equity in public health programs and the implications for our most vulnerable populations. A one-size-fits-all approach to health initiatives does not best serve these populations, especially those living in rural areas with limited transportation options. We also highlight the ingenuity, sacrifice, and creativity employed by public agency administrators to meet their professional responsibility to social equity and enhance programmatic outcomes. This example aligns with the familiar adage of public servants doing more with less to meet the needs of their communities. Despite its heavy reliance on transportation, the Pennsylvania COE program is a success because of the unwavering commitment of public health leaders and medical partners. It is, therefore, unsurprising that the number one recommendation agency respondents identified to improve transportation inequity in OUD treatment was increased collaboration and coordination between community partners. Money alone is an insufficient means to an end of transportation equity in opioid treatment options. Collaboration, flexibility, and coordination are vital in promoting equitable outcomes in public health initiatives.

Through this research, we seek to paint a holistic picture of the administration challenges overcome by public health leaders when state expectations fall short of socially just outcomes. Policy developers and funders must increase the opportunity for flexibility and responsible customization into program guidance so that administrations can act with authority and autonomy to meet the unique needs of their communities. Without this flexibility, the public service adage of doing more with less and reliance on sacrificial efforts will continue to erode the effectiveness of public health efforts.

## Figures and Tables

**Table 1:** COE Transportation Equity Survey: survey topics by version.

**Agency-Level Demographics (Identical for all three survey versions)**
Respondent Title
COE Agency Name
Designation Tenure
# of Clients with OUD Served Annually
OUD-related Services Offered (Select all that Apply)
Client Demographic Estimates (Race/Ethnicity, Age, and Gender)
In 2019, Pennsylvania Opioid Use Disorder Center of Excellence funding switched from grant funded to a Per-Member-Per-Month reimbursement. Did this change in payment structure impact your agency’s ability to provide sufficient transportation services to clients with opioid use disorder?
In considering the your Per-Member-Per-Month rate as a Center of Excellence, how well does the program support your agency’s ability to adequately fund all components of its opioid use disorder treatment activities?
Which land use category best describes the location(s) where clients or patients make in-person visits to your agency?
**Transportation Equity Questions (Identical Wording on all three survey versions. For the Multiple Service Sites with different Land-Uses Version, the question set is repeated for each land-use category with instructions to focus on one land-use category at a time).**
In your role, are you aware of the U.S. Code 5311 Formula Grants Other Rural Areas federal grant program which aims to provide and enhance the access of people in non-urbanized areas to health care, shopping, education, employment, public services, and recreation?
In what PA County are the sites/site located?
How important is the consideration of transportation needs when referring and/or enrolling individuals with opioid use disorder into care?
Which of the following transportation supports, if any, does your agency provide to clients with opioid use disorder? Select all that apply.
Among the individuals with opioid use disorder served by your agency, please rank the most widely observed modes of transportation for accessing care
Based on your observations, how difficult is it for individuals with opioid use disorder served by your agency to get to their treatment-related appointments?
What are the most commonly observed transportation challenges? (Check ALL that apply)
On average, what percentage of individuals with opioid use disorder served by your agency (per week) do you observe missing appointments due to transportation barriers?
Which of the following initiatives might benefit infrastructure or transportation services for individuals with opioid use disorder served by your agency? (Check ALL that apply)

**Table 2:** COE Transportation Equity Survey: consideration of transportation needs (all agencies = 23; rural = 7; suburban = 7; urban = 9).

Responses	All Agencies	Rural	Suburban	Urban
Not at All	n = 1 (4.3%)	n = 0 (0%)	n = 0 (0%)	n = 1 (11.1%)
Slightly Important	n = 1 (4.3%)	n = 0 (0%)	n = 0 (0%)	n = 1 (11.1%)
Important	n = 2 (8.7%)	n = 0 (0%)	n = 1 (14.3%)	n = 1 (11.1%)
Very Important	n = 9 (39.1%)	n = 3 (42.9%)	n = 5 (71.4%)	n = 1 (11.1%)
Critical	n = 10 (43.5%)	n = 4 (57.1%)	n = 1 (14.3%)	n = 5 (55.6%)

**Table 3:** COE Transportation Equity Survey: PMPM rate satisfaction (all agencies = 23; rural = 7; suburban = 7; urban = 9).

Responses	All Agencies	Rural	Suburban	Urban
Not Well	n = 1 (4.3%)	n = 0 (0%)	n = 0 (0%)	n = 1 (11.1%)
Slightly Well	n = 4 (17.4%)	n = 2 (28.6%)	n = 1 (14.3%)	n = 1 (11.1%)
Moderately Well	n = 8 (34.8%)	n = 2 (28.6%)	n = 2 (28.6%)	n = 4 (44.4%)
Very Well	n = 9 (39.1%)	n = 3 (42.9%)	n = 4 (57.1%)	n = 2 (22.2%)
Extremely Well	n = 1 (4.3%)	n = 0 (0%)	n = 0 (0%)	n = 1 (11.1%)

**Table 4:** COE Transportation Equity Survey: ease of appointment access (all agencies = 23; rural = 7; suburban = 7; urban = 9).

Reponses	All Agencies	Rural	Suburban	Urban
Extremely Easy	n = 1 (4.3%)	n = 0 (0%)	n = 0 (0%)	n = 1 (11.1%)
Easy	n = 3 (13.0%)	n = 1 (14.3%)	n = 1 (14.3%)	n = 1 (11.1%)
Neither Easy nor Difficult	n = 6 (26.1%)	n = 2 (28.6%)	n = 1 (14.3%)	n = 3 (33.3%)
Difficult	n = 13 (56.5%)	n = 4 (57.1%)	n = 5 (71.4%)	n = 4 (44.4%)

## Data Availability

The original contributions presented in the study are included in the article; further inquiries can be directed to the corresponding author.
